# Unlock the Thermogenic Potential of Adipose Tissue: Pharmacological Modulation and Implications for Treatment of Diabetes and Obesity

**DOI:** 10.3389/fendo.2015.00174

**Published:** 2015-11-19

**Authors:** Xiao-Rong Peng, Peter Gennemark, Gavin O’Mahony, Stefano Bartesaghi

**Affiliations:** ^1^Cardiovascular and Metabolic Diseases IMED Biotech Unit, Diabetes Bioscience Department, AstraZeneca R&D, Mölndal, Sweden; ^2^Cardiovascular and Metabolic Diseases IMED Biotech Unit, Drug Metabolism and Pharmacokinetics Department, AstraZeneca R&D, Mölndal, Sweden; ^3^Cardiovascular and Metabolic Diseases IMED Biotech Unit, Medicinal Chemistry Department, AstraZeneca R&D, Mölndal, Sweden

**Keywords:** brown adipose tissue, thermogenesis, uncoupling protein 1, drug discovery, PPARγ agonists, thiazolidinediones, β_3_-adrenergic receptor agonists, FGF21 analogs

## Abstract

Brown adipose tissue (BAT) is considered an interesting target organ for the treatment of metabolic disease due to its high metabolic capacity. Non-shivering thermogenesis, once activated, can lead to enhanced partitioning and oxidation of fuels in adipose tissues, and reduce the burden of glucose and lipids on other metabolic organs such as liver, pancreas, and skeletal muscle. Sustained long-term activation of BAT may also lead to meaningful bodyweight loss. In this review, we discuss three different drug classes [the thiazolidinedione (TZD) class of PPARγ agonists, β_3_-adrenergic receptor agonists, and fibroblast growth factor 21 (FGF21) analogs] that have been proposed to regulate BAT and beige recruitment or activation, or both, and which have been tested in both rodent and human. The learnings from these classes suggest that restoration of functional BAT and beige mass as well as improved activation might be required to fully realize the metabolic potential of these tissues. Whether this can be achieved without the undesired cardiovascular side effects exhibited by the TZD PPARγ agonists and β_3_-adrenergic receptor agonists remains to be resolved.

## Introduction

According to the International Diabetes Federation (IDF), 8.3% of adults worldwide – 370 ­million people – have type-2 diabetes (T2D), and the number of people with the disease is set to rise beyond 592 million in under 25 years (1). Although there are many drugs available for diabetes, none of them safely and durably prevent or reverse disease progress and its associated comorbidities. Poor diet, sedentary lifestyle, and obesity are considered major risk factors for diabetes. Inappropriate fuel handling by adipose tissue, liver, and skeletal muscle, combined with ectopic lipid deposition in key metabolic organs (such as liver, pancreas, muscle, and heart) have been hypothesized to play a significant role in the development of insulin resistance. Insulin resistance increases the overall burden on β-cells, which over time leads to β-cell failure and development of T2D.

Adipose tissue can be grossly divided into two major depots, white adipose tissue (WAT) and brown adipose tissue (BAT). WAT stores excess energy as triglycerides (TGs), which can be mobilized by lipolysis to generate free fatty acids (FFAs) for use by other tissues. BAT, on the other hand, is the main site of non-shivering thermogenesis (NST), which requires a brown adipocyte-specific protein called uncoupling protein 1 (UCP1).

Non-shivering thermogenesis by BAT is an interesting target for the treatment of metabolic disease due to the high metabolic capacity of BAT. BAT is highly vascularized and richly innervated by sympathetic nerves, and its activation is predominantly regulated by the sympathetic nerve system via β-adrenergic receptors (β-ARs). Enhancing energy expenditure (EE) through activation of NST by β_3_-adrenergic receptor (β_3_-AR) agonists has been investigated as an alternative to inhibition of food intake for bodyweight loss. This has, however, been unsuccessful in human clinical trials. This lack of effect on EE was partly attributed to negligible BAT function in adult humans compared to the situation in rodents.

**Figure 1:**
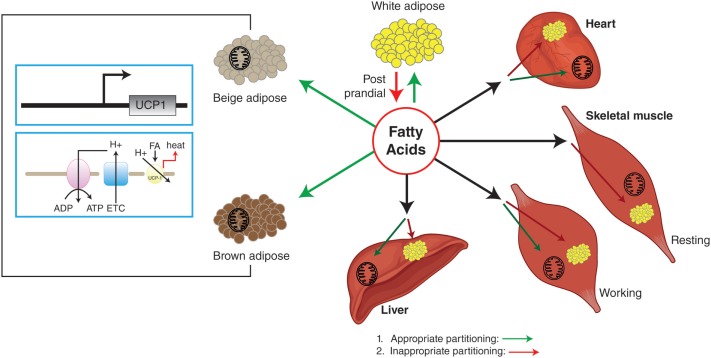
**White adipose tissue stores excess energy as triglycerides that can be mobilized by lipolysis to generate FFA for use by other tissues**. BAT is the main site of NST, which is carried out by UCP1. Beige adipocytes have uncoupling capabilities similar to brown adipocytes, but are found in what is normally considered WAT. Appropriate partitioning and oxidation of fatty acids into BAT, WAT, beige adipocytes, and other metabolic organs can reduce ectopic fat deposition in metabolic organs, resulting in improved insulin sensitivity. Green arrows indicate appropriate partitioning and red arrows indicate inappropriate partitioning.

The rediscovery of BAT in the adult human in 2007, and the subsequent demonstration of functional involvement of human BAT in NST have revitalized this area (2–5). In addition, the presence of brown-like adipocytes in WAT [referred to as beige or brown-in-white (brite) adipocytes] further increased the interest in brown adipocyte biology, as WAT mass is relatively large and any increase in cellular energetics in this tissue may have a significant impact on whole-body metabolism and EE. The beige nomenclature will be used for this review. Utilization of FFAs during NST could lead to depletion of brown and or beige adipocytes’ lipid stores, which may result in redistribution of fuels [including glucose and non-esterified fatty acids (NEFA)] toward brown and beige adipocytes. In turn, this could lead to a reduced fuel over-supply to other metabolic organs (heart, skeletal muscle, and liver) and, thus, improved insulin sensitivity (Figure 1).

Transcriptional and hormonal regulation of the “browning” program of adipose stem cells and characterization of the molecular signature have been reviewed extensively elsewhere (6–12). This paper focuses on the metabolic potential of BAT and beige adipocytes, how these systems can be manipulated by pharmacological means, and how to assess if a brown adipocyte phenotype has been achieved by pharmacological intervention. Finally, we discuss the challenges of drug discovery in this area by reviewing three classes of clinically investigated pharmacological agents that regulate various aspects of BAT and beige adipocyte function: thiazolidinedione (TZD) PPARγ agonists, β_3_-AR agonists, and fibroblast growth factor 21 (FGF21) analogs.

## Brown Adipose Tissue in Adult Humans

Typically, BAT is located in the interscapular (iBAT), cervical, auxiliary, perirenal and paraaortic areas of animals or human infants (13–15). In adult humans, BAT depots have a diffuse anatomic distribution, with mixtures of white and beige adipocytes, seeming to coexist in close proximity. Beige adipocytes have also been reported to arise in what are normally considered WAT depots (such as inguinal adipose in rodents) in response to various stimuli such as cold, TZDs, and β-AR agonists (9). In humans, beige adipocytes were found in the WAT of pheochromocytoma patients due to the presence of catecholamine-secreting tumors (16, 17) and in the subcutaneous adipose of severely burned patients where heat loss is increased and who experience prolonged adrenergic stress (18). Both lineage-tracing studies and transcriptional profiling of classical brown and beige adipocytes indicate that these two cell types seem to originate developmentally from distinct cell lineages. Classical brown adipocytes in interscapular BAT arise from precursors that are *myf5*^+^, a gene known to be also expressed in committed skeletal muscle precursors (19, 20). The developmental origin of the beige adipocyte remains to be elucidated. The whole-body NST will have contributions from both classical brown and beige adipocytes.

It is now beyond doubt that BAT is present in adult humans and plays a role in NST (3). BAT activity can be detected by ^18^F-fluorodeoxyglucose (^18^F-FDG) uptake using PET–CT (2, 4, 5). Retrospective analysis of populations that have undergone PET–CT examination indicates that the prevalence of BAT varies between 1 and 5%. For example, one study on 4011 asymptomatic individuals (<5% obese subjects) showed that BAT prevalence is 5% in female and 1.3% in male (21). The BAT-positive subjects had lower body mass index (BMI), less visceral and subcutaneous fat areas, lower fasting glucose and TG levels, and increased HDL cholesterol concentrations compared to the BAT-negative subjects. Similar results were reported for 56 healthy volunteers (22), and for cancer patients (2, 23). The inverse correlation between BAT activity and BMI was further confirmed in non-diabetic subjects over a wide range of body compositions (BMI ranging from 22 to 48 kg/m^2^) (4, 24–27). A recent retrospective analysis of ^18^F-FDG uptake data (analysis of the neck regions of two relatively large cohorts of individuals) reports that the average body weight of BAT-positive individuals is approximately 5 kg lower compared to that of BAT-negative subjects (21, 23).

Using a combination of MRI and molecular analysis, Enerbäck’s group clearly demonstrated that iBAT in human infants consists of classical brown adipocytes (15). However, the molecular signature of brown adipocytes isolated from the neck regions of adult humans resembles that of the rodent beige phenotype rather than classical brown adipocytes (15, 28–31). What causes the BAT phenotype transformation between infant and adult humans is not understood. It is conceptually important to unravel the function, regulation, and differentiation of beige and classical brown adipocytes in order to be able to pharmacologically enhance thermogenesis in humans. For example, if the adipocytes with various white and beige appearances in adult human neck region are, in fact, simply dormant brown adipocytes, they may be readily re-activated by cold or sympathomimetics. In addition, it is important to understand the translational aspects of BAT biology, i.e., whether the same or different pharmacological agent(s) will show desirable effects in preclinical animal species and man?

## Bat and Metabolic Significance

The contribution of BAT to whole-body metabolism in rodents has recently been examined using tools such as radioactive tracers and PET–CT imaging. Bartelt et al. showed that BAT is the major site of triglyceride-rich lipoprotein (TRL) clearance during acute cold exposure (32). Cold exposure also dramatically increased the glucose disposal to BAT tissue. The remarkable capacity of BAT to take up substrates is illustrated by the ratio of BAT mass to the total glucose and TG uptake by BAT compared to that of other major organs in mice under cold challenge. Labbe et al. extended this observation through PET–CT analysis of the rate of substrate flux and oxidation in the iBAT of both warm- and cold-adapted rats (33). The rate of glucose uptake into iBAT was relatively low at 27°C but increased 10-fold upon acute cold exposure and increased 46-fold after cold acclimation at 10°C. Similarly, NEFA levels rose 6-fold upon acute cold exposure, and ~100-fold after cold acclimation. The metabolic activity of the iBAT reached levels similar to that of heart and liver after 6 h of cold exposure. In spite of these results, it should be kept in mind that although BAT glucose uptake per unit volume of tissue is important, the bulk of glucose turnover during cold exposure is mediated by skeletal muscle metabolic activation even when shivering is minimized (7).

It is more challenging to determine the specific contribution of beige adipocytes to whole-body metabolism. Bartelt et al. showed that acute cold exposure also increases TRL uptake in inguinal WAT (iWAT), but to a smaller extent compared to iBAT (32, 34). Seale’s group reported an aP2-PRDM16 transgenic mouse that exhibited a highly favorable metabolic phenotype, in which iBAT remained unchanged but with extensive browning in iWAT. This suggests that beige adipocytes may contribute to the overall metabolic phenotype observed in this mouse (35).

**Figure 2:**
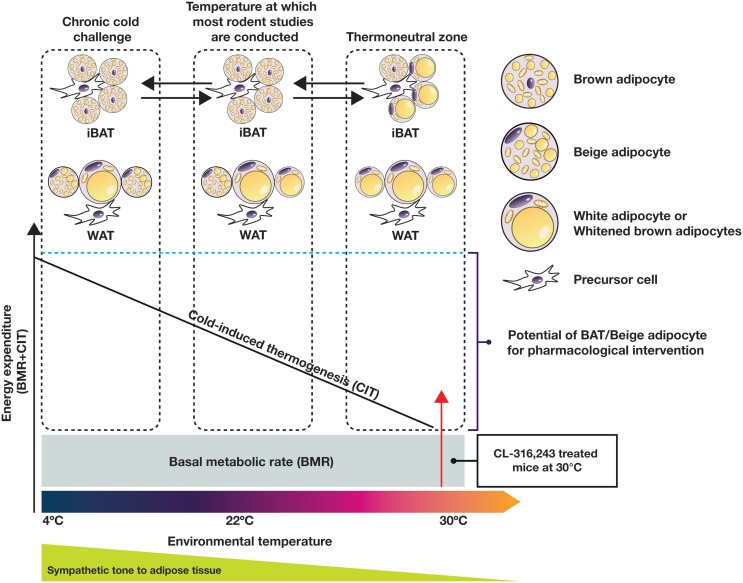
**Adaptation of EE and adipose tissue morphology and function to the changes of environmental temperature in the mouse**. Sympathetic tone to adipose tissue plays an important role in this process. Most current published rodent studies are conducted at temperatures below the thermoneutral zone, while humans typically live near the thermoneutral zone. For adequate translation between species, rodent studies should be performed under thermoneutral conditions.

On the whole-body level, Reitman’s group dissected the relative contributions of cold-induced, diet-induced, and physical activity-associated EE in mice in relation to the basal metabolic rate (BMR) at various temperatures (36). This work clearly illustrated that at 22°C, the temperature at which most reported metabolic studies have been conducted, mice expend a relatively large amount of energy to generate heat (120% of BMR). Adult humans, on the other hand, live in or near their thermoneutral zone, with a relatively small contribution from adaptive thermogenesis to EE (5% of BMR) (11). Interestingly, in mice housed at thermoneutrality (30–32°C), the relative contributions of BMR, diet, physical activity, and adaptive thermogenesis in mice to overall EE are reported to be ~60, 12, 25, and 0%, respectively (36). These figures are very similar to the relative contributions seen in humans with low activity levels, and might represent experimental conditions more suitable for translational research in this field. The EE increase in relation to external temperature is conceptually depicted in Figure 2 (not scaled to real data).

Numerous studies that address the role of human BAT activation and its quantitative impact on whole-body metabolism have been published. Based on the heat production capacity of mouse BAT, Rothwell and Stock proposed in the 1980s that 40–50 g of BAT, if maximally activated, could account for 20% of daily EE in human (37). More recently PET–CT measurements estimated the average active BAT volume in healthy humans to be 137 cm^3^, corresponding to a conservative estimate of around 50 g BAT mass (11). Virtanen et al. estimated the EE of human BAT to be 55 W/kg (5) based on the rate of glucose uptake during cold exposure, as measured by dynamic PET–CT. These conservative estimates suggest that the EE of fully activated BAT could amount to 2–5% of BMR. Recent cold exposure experiments confirmed that the cold-induced NST-associated increase in EE accounts for 0–15% of BMR (11). Using a human body-composition model (38), we further extrapolated that a 4% increase of BMR could lead to a 3% bodyweight reduction per year, assuming that the effects are sustained. A key assumption in such an extrapolation is that functional tolerance can be avoided (39). In support of this assumption, some pieces of evidence suggest that increased thermogenesis is not always fully compensated for and negated by an increase in food intake, for reasons that are not fully understood (40).

Cypess et al. recently reported a 13% increase in BMR as a result of BAT activation upon administration of an acute dose of the β_3_-AR agonist mirabegron (41). Again, assuming that this BMR increase could be sustained upon chronic treatment, modeling suggests that this could potentially lead to an 8% bodyweight loss per year in BAT-positive healthy men. Besides potential functional tolerance, it remains uncertain if chronic β-AR-mediated stimulation of BAT is possible without encountering the side effects often associated with β-AR agonists.

Bodyweight reduction has been shown to have positive effects not only on preventing the progression of pre-diabetes to diabetes, but also leads to a reduction of hemoglobin A1c (HbA1c) (42). More recently, Hanssen et al. showed that 10-day cold acclimation in obese T2D patients could increase BAT activity, which was in turn associated with a reduction of BAT TG content and increased EE (43). One very encouraging observation is the 43% improvement of glucose infusion rate during a clamp study. Importantly, the improvement in insulin sensitivity of both adipose and skeletal muscle appeared before any bodyweight change could be seen (43). As indicated by Hanssen et al., the cold-induced improvement in insulin sensitivity exceeds that which was observed after long-term exercise training. To place this in a pharmacological context, this improvement in insulin sensitivity is similar in extent to that seen after 2-week treatment with dapagliflozin (Farxiga/Forxiga), which affords an 18% improvement in tissue glucose disposal (44).

## Functional Brown/Beige Adipocytes

The remarkable metabolic capacity of classical brown adipocytes in the activated state has been well characterized using rodent brown adipocytes. Functional characterization of beige adipocytes from the inguinal adipose tissue of mice or differentiated beige adipocytes from human adipose stem cells suggests that these cells are functionally similar to classical brown adipocytes (45, 46). The developmental origin of brown and beige adipocytes is currently an area of intense research and has been extensively reviewed elsewhere (47, 48).

**Figure 3:**
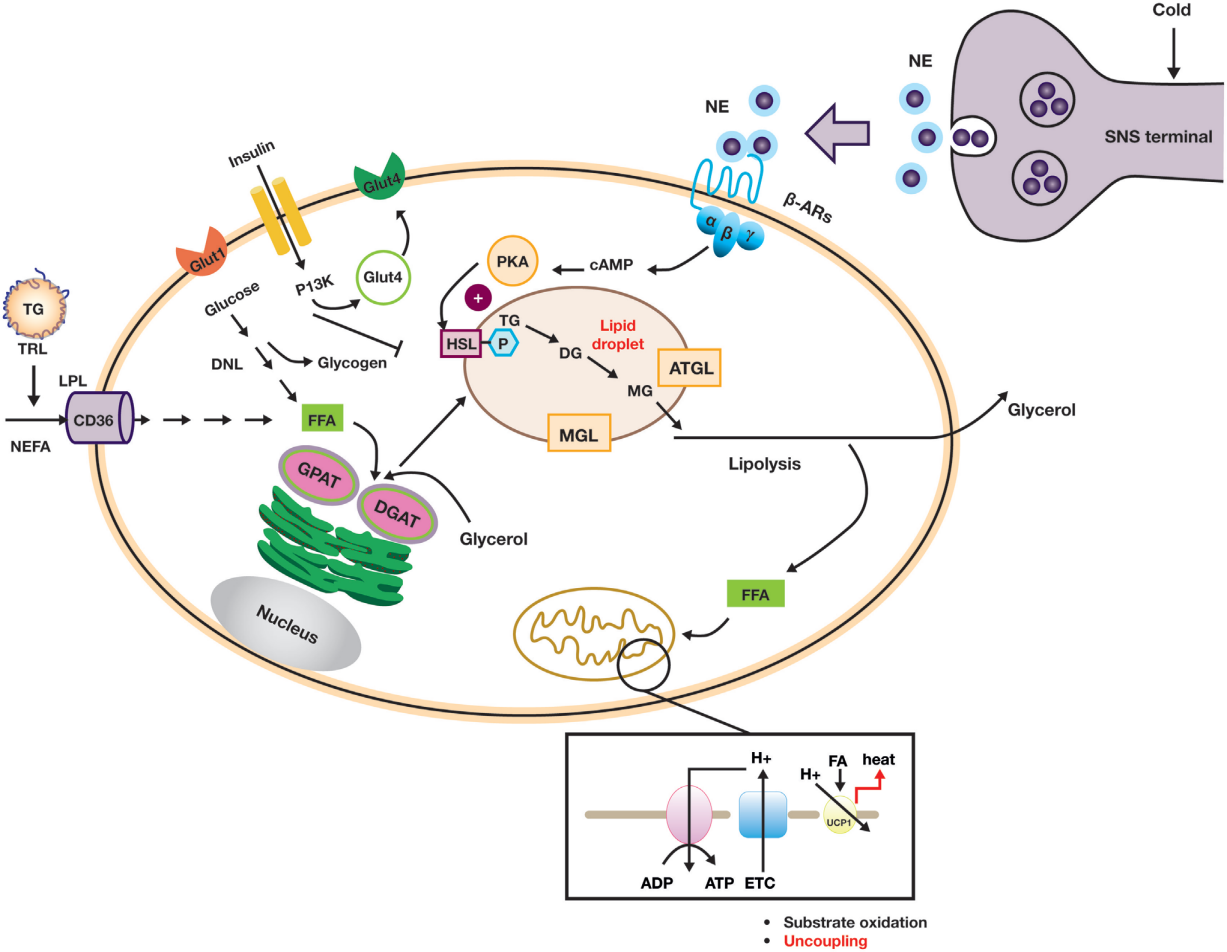
**Diagram showing key metabolic and signaling pathways in brown or beige adipocytes**.

Uncoupling of the mitochondrial transmembrane proton gradient in brown adipocytes in order to generate heat in response to cold is a complicated but well-orchestrated event. The key metabolic and signaling pathways in the brown or beige adipocyte are summarized in Figure 3. To be able to carry out this function, brown adipocytes need to be equipped with a complex machinery that is able to (i) signal through norepinephrine (NE) or other catecholamines via β-ARs, (ii) generate intracellular FFAs through hydrolysis of TGs from lipid droplets (lipolysis), and (iii) uncouple the ATP-generating process via UCP1 activation (13, 49). Chronically, cold challenge leads to a recruitment process in which cell proliferation, mitochondrial biogenesis, and angiogenesis are enhanced. Given the broad range of cellular processes that are involved in these events, it is not surprising that large numbers of genes/factors have been described to regulate the differentiation and function of brown and beige adipocytes (9, 34).

The β_3_-AR has emerged as a leading molecular target for the activation of brown or beige adipocytes (18, 41). Rodent and human adipose tissue display different β-AR expression profiles. (50–53). In mouse, the β_3_-AR is highly expressed in both BAT and WAT with several-fold higher abundance than the β_1_-AR, whereas in the adult human, the β_3_-AR is only expressed in BAT. In human WAT, β_1_-AR has been reported to be 50-fold more abundant than β_3_-AR (52). The β_3_-AR subtypes also differ in their potency toward various ligands, G-protein coupling, and desensitization. The binding affinity of NE to β_3_-AR was reported to be in the low micromolar range, while the potency for cAMP accumulation is in the low nanomolar range when measured in intact cells, suggesting that the coupling efficacy to adenylyl cyclase of β_3_-AR was higher than that of β_1_-AR (52, 54). In addition, β_1_-AR desensitizes more rapidly than β_3_-AR upon exposure to agonists. This led to the hypothesis that β_1_-AR may mediate the NE response to low levels of sympathetic stimulation. On the other hand, the activation of β_3_-AR may require higher levels of sympathetic stimulation, but once activated this receptor is likely to deliver a more sustained effect (52). It is important to note that circulating NE levels are generally in the low nanomolar concentration range, and it is likely that NE concentrations at the synaptic clefts will be much higher during cold response, providing sufficiently high local concentrations in BAT to enable local activation of β_3_-AR in spite of the low plasma concentration. Recently, several studies have shown that systemic administration of adrenergic activators, such as isoproterenol (ISO) and ephedrine, fail to elicit BAT activation in man (55–57). To properly interpret these clinical data, it is crucial to understand if the plasma concentrations of the adrenergic agonists used reached a sufficiently high concentration to enable activation of lipolysis and thermogenesis in human BAT.

Norepinephrine-mediated β-AR activation results in an increased intracellular cAMP concentration, which in turn stimulates lipolysis in brown adipocytes via activation of the protein kinase A pathway. Lipolysis is a stepwise process with different enzymes acting at each step: TGs are hydrolyzed by desnutrin/adipose triglyceride lipase (ATGL) to form diacylglycerol (DAG). DAG is then hydrolyzed by hormone-sensitive lipase (HSL) to monoacylglycerol and, subsequently glycerol, with a fatty acid released at each stage. Intracellular FFAs are the direct activators of UCP1 (58). In humans, the BAT radiodensity (which is indicative of intracellular TG stores) is inversely correlated with NST, strongly suggesting that depletion of intracellular TG occurs during cold exposure. In rats, inhibition of lipolysis by nicotinic acid-mediated GPR109a agonism significantly reduced the oxidative capacity of iBAT in response to cold, again suggesting a key role for lipolysis in UCP1-mediated thermogenesis (33). Additionally, adipose tissue-specific knockout of ATGL led to the formation of “whitened” brown adipocytes and resulted in impaired lipolysis and defective thermogenesis in BAT (59–62).

The brown adipocytes’ cold-depleted energy stores are replenished by *de novo* lipogenesis and glycogen synthesis, which requires cellular uptake of circulating glucose and FFAs (derived either from TRLs or from lipolysis in WAT). In brown adipocytes, glucose uptake is mostly mediated by glucose transporter 1 (GLUT1) and glucose transporter 4 (GLUT4) and subsequently stored as glycogen or converted to lactate through anaerobic glycolysis. As shown in Figure 3, FFAs are transported into the cell by cluster of differentiation 36 (CD36) and TGs are subsequently synthesized through re-esterification by a series of enzymes, including glycerol-3-phosphate acyltransferase (GPAT) and diacylglycerol O-acyltransferases (DGATs) (49). The process of recruitment of new brown or beige adipocytes and the maintenance of mature adipocyte function during acute and chronic cold acclimation are subject to complex transcriptional and hormonal regulation, for example, by PPARγ, bone morphogenic proteins (BMPs), the thyroid axis, and FGF21. These regulatory mechanisms have been extensively reviewed elsewhere (8, 47, 48, 63).

The functional activity of BAT is reduced by chronic warm acclimation, old age, obesity, and diabetes. Rodent models of genetic deficiency of leptin (*ob/ob*, *db/db*, and obese *fa/fa* rats) are cold sensitive, and their brown adipocytes have a white adipocyte appearance with reduced expression of UCP1 protein (64, 65). Old age and lack of cold challenge also reduce the thermogenic activity of the brown adipocytes (66). Brown adipocytes isolated from guinea pigs housed at 30°C appear unilocular, although these cells appear to retain their brown adipocyte identity and are able to respond to NE with a robust increase of thermogenesis, which is not the case for adipocytes isolated from WAT (67). In humans, BAT activity is inversely correlated with age, fat mass, and BMI. Insulin-stimulated glucose uptake is also compromised in the BAT of diabetic individuals (68). Recent reports that both chronic cold acclimation and weight loss can enhance BAT activity in humans are encouraging (27, 69, 70), indicating that reduced BAT function may be restored. In this respect, adipocytes or adipose precursor cells are highly plastic and able to adapt to the functional needs, as has been shown in rodent using lineage tracing experiments (71).

## Thiazolidinediones

The TZDs [represented by rosiglitazone (Avandia) and pioglitazone (Actos)] are a chemical class of PPARγ agonists used as insulin sensitizers for the treatment of T2D (72). The primary site of action of the TZDs is adipose tissue, where they improve several functional aspects, including the uptake and storage of plasma NEFA. They also increase FFA mobilization under fasting conditions and enhance postprandial suppression of FFA mobilization by insulin (73).

Thiazolidinediones have been shown to increase UCP1 expression and BAT mass in rodents. Rosiglitazone has been shown *in vitro* by several laboratories to induce UCP1 in rodent brown adipocytes and differentiated adipose stem cells (74–77). In addition, chronic treatment of human subcutaneous adipose stem cells with rosiglitazone upregulated several components of the mitochondrial electron transport chain, which is consistent with what has been observed in human (78). The UCP1 protein in human adipocytes differentiated *in vitro* in the presence of rosiglitazone is only functional when cells have been allowed to differentiate for a longer time than is typically reported in mouse studies (45). Although TZDs have the ability to recruit the “browning program” in both mouse and human adipocytes, the thermogenic capacity of UCP1-expressing cells cannot be unleashed without subsequent activation (e.g., by β-AR agonists). An increase in the oxygen consumption rate (OCR) of human adipocytes differentiated *in vitro* in the presence of rosiglitazone could only be observed when the cells were stimulated with isoproterenol or in the presence of exogenously provided FFAs. The increase of OCR in these beige cells is completely UCP1 dependent, as UCP1 knock-down abolishes the effect (45).

Various *in vivo* rodent models of insulin resistance have been used to show that the TZDs increase UCP1 mRNA in iBAT and overall iBAT weight. However, this is not associated with a subsequent increase in thermogenesis or whole-body EE. In addition, TZD treatment leads to the brown adipocytes becoming lipid filled (79–81). This led to the hypothesis that obese and diabetic animals or humans could first be primed by a PPARγ agonist to expand the BAT capacity, followed by its activation via a β-AR agonist. This concept was tested pre-clinically in *ob/ob* mice by pre-treating with a PPARγ agonist (COOH, a non-TZD PPARγ agonist) followed by treatment with the β_3_-AR agonist CL-316,243. Synergistic effects on EE and bodyweight reduction were indeed observed in this single study (82). However, a later study using rosiglitazone followed by acute cold exposure failed to reproduce these effects (83). A possible explanation for the lack of increased EE in spite of increased TG storage, UCP1 expression and BAT mass induced upon PPARγ activation by rosiglitazone (84) may be the downregulation of β_3_-AR and iodothyronine deiodinase type II (DIO2) expression caused by TZD treatment (85, 86). In addition, as COOH belongs to a different structural class of PPARγ agonists to the TZDs, it is not unlikely that different outcomes could be observed between the two compounds. In humans, 12 weeks of pioglitazone treatment has been shown to generate a small increase in UCP1 mRNA levels in subcutaneous adipose tissue (87). However, combined pioglitazone and ephedrine treatment for 12 weeks in obese human subjects failed to deliver significant bodyweight reduction (88).

When comparing the observed outcomes of studies employing PPARγ agonists, it must be kept in mind that even structurally closely related compounds may exhibit very different PPARγ-dependent pharmacodynamic profiles. This is, in part, due to ligand-dependent modulation of the PPARγ protein that leads to recruitment of different coactivator and corepressor proteins, in turn resulting in a unique transcriptional profile for each compound (89). Since the identification of PPARγ as the molecular target of the TZDs (90), significant effort has been invested by the pharmaceutical industry in PPARγ agonist drug discovery (91–96) albeit largely without commercial success (97). The availability and high throughput of PPARγ LBD-based ligand binding assays and chimeric PPARγ–GAL4 reporter gene/transactivation assays enabled the generation of many highly potent, structurally diverse, selective PPARγ full and partial agonists. Despite this, since the launch of pioglitazone and rosiglitazone, no new selective PPARγ agonists have survived clinical testing and remain on the market, mainly due to preclinical and clinical safety issues (98–101). The central role of PPARγ in adipose biology has, however, not diminished due to these failures and PPARγ remains an attractive but challenging drug target.

The lack of translation between *in vitro* receptor binding and the functional (both *in vitro* and *in vivo*) effects of the compounds and inter-species differences in PPARγ biology are considered major obstacles in this field. This is due to a number of factors, including the combinatorial nature of the activation of the PPARγ:RXR heterodimer (102, 103), subtle differences in coactivator/corepressor recruitment between superficially related compounds (104–107), ligand effects on the extent of posttranslational modifications (108, 109), and non-transcriptional effects of the ligands (72). This suggests that a reductionist approach to PPARγ agonist discovery based on the use of isolated protein domains and chimeric reporter gene assays is unlikely to provide compounds with the desired functional or clinical outcome.

Since the “traditional” approach of optimizing receptor binding and agonist potency has not borne fruit, a radical change in the preclinical approach to PPARγ drug discovery is needed in order for the functional potential (for example, browning of WAT) of small-molecule PPARγ activation to be realized. For example, the application of phenotypic screening in relevant cell systems [i.e. primary human cells (110)] is one approach to frontload the functional assessment of compounds, with traditional *in vitro* assessment of PPARγ activity included in a secondary wave of assays. In addition, the recent widespread availability of omics techniques (such as *RNA*omics and proteomics) makes the preclinical identification of PPARγ agonists with a desirable functional profile a realistic prospect. A combination of such approaches avoids focus on a single receptor-dependent pathway or mechanism and allows pleiotropy to be accounted for. However, whether the perceived target-related risk associated with PPARγ agonism is considered acceptable in proportion to the potential commercial viability of a safe PPARγ agonist remains to be seen.

## Beta-3 Adrenergic Receptor

Several sympathomimetic β_3_-AR agonists that selectively stimulate rodent brown and white adipocyte lipolysis were discovered from the mid-1980s onward (e.g., BRL-37344, CL-316,243, and CGP-12177A) (50, 51, 111). Early optimization of these compounds was mostly performed in rodent tissue or cell models, as the human β_3_-AR was not cloned until 1989 (112). The compounds showed potent anti-obesity and anti-diabetic effects in rodent models of obesity and diabetes, but none of these compounds advanced beyond the clinical phase II due to lack of efficacy. Specifically, compounds optimized using rodent β_3_-AR did not effectively translate to human. Several β_3_-AR agonists were synthesized and evaluated after the cloning of human β_3_-AR cDNA (113). A summary of studies investigating the effect of β_3_-AR agonism on EE in man is given in Table 1. Note that many binding and adenylyl cyclase activity assays were performed using isolated membrane preparations, returning β_3_-AR binding affinities in the micromolar range for the compounds tested. Lower potencies (in the nanomolar range) have been reported for the same compounds when measured in whole-cell assays (cAMP, lipolysis, or respiration) (52), suggesting a G-protein coupling efficiency for the β_3_-AR that is only captured in a whole-cell context.

**Table 1:** ***In vitro* properties of β_3_-adrenergic receptor agonists that have been tested in humans, and their effect on energy expenditure in man**.

PHYSICAL AND PHARMACOKINETIC PROPERTIES						
**Compound name(s) and chemical structure**	**CL-316,243** 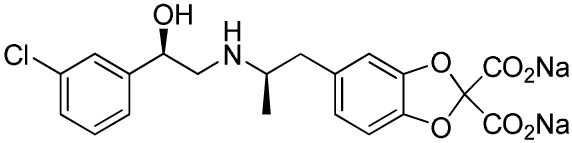	**ZD7114** 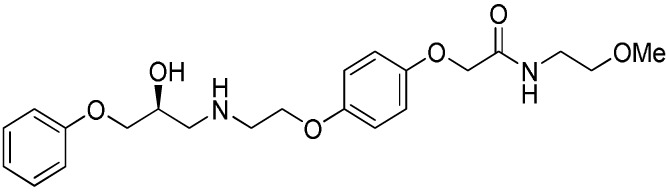	**ZD2079 (Talibegron)** 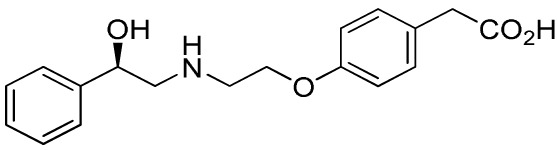	**L-796568** 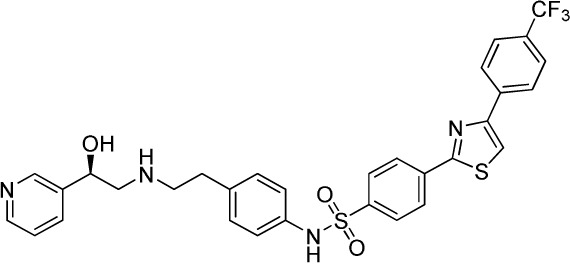	**TAK-677 (Rafabegron)** 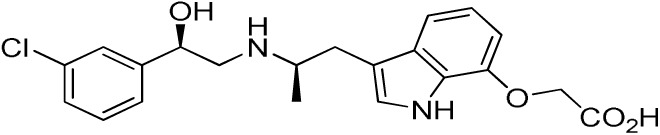	**Mirabegron (YM-178)** 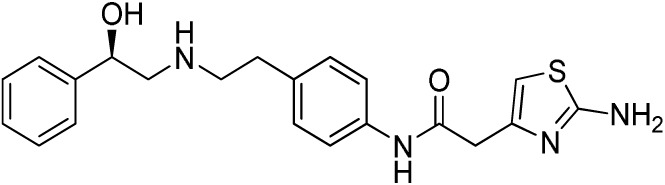
**MW (g/mol)**	466.8 (Na_2_ salt), 421.8 (free acid)	418.5	315.4	624.7	402.9 (free acid)	396.5
**clogP**	−1.4	2.1	−0.3	5.3	0.7	1.3
**Human β_3_ potency/TE**	EC_50_ 1.15 μM/0.63^a^ (114)	*K*_act_ 20 nM (for ZD201651, the major active metabolite) (115)	K_act_ 191 nM/0.91^a^ (116)^b^	3.6 nM/0.94^a^ (117)	EC_50_ 0.062 nM/1.16^a^ (118)	22 nM/0.8^a^ (119)
**Rodent β_3_ potency/TE**	Rat *K*_i_ 1 μM, *K*_act_ 0.71 nM/1^a^ in β_3_ overexpressing CHO cells (111)	–	–	–	0.016 nM/1.1^a^ (rat cAMP)	–
**Fold selectivity β_3_:β_2_:β_1_**	1:228:96 Based on CHO data (114). Reported as an antagonist @ β_1_ and β_2_ (120)	–	–	1:667 (partial):1333 (partial)	1:209 (partial):1032 (partial)	1:>446 (partial):>446 (partial)
***T*_1/2_/%F**	16 h/10% (human)^c^	–	–	>8 h/17% (rat)	–	–
**PHARMACODYNAMICS**						
**Population**	Healthy young lean males (124)	Obese men and women with BMI 27–39 kg/m^2^ (115)	Obese men and women with BMI 27–39 kg/m^2^ (115)	Healthy overweight to obese men (121)	Obese men and women, mean BMI 33.9 kg/m^2^ (122)	Healthy male subjects with detectable BAT (41)
**Administration**	1.5 g/day for 8 weeks (*n* = 10)	150 mg/day for 2 weeks (*n* = 5)	1.2 g/day for 2 weeks (*n* = 9)	375 mg/day for 28 days (*n* = 10)	0.5 mg BID for 29 days (*n* = 22)	200 mg acutely (*n* = 12; crossover study)
	Placebo (*n* = 4)	300 mg/day for 2 weeks (*n* = 8) Placebo (*n* = 22)	Placebo (*n* = 8)	Placebo (*n* = 10)	Placebo (*n* = 4)	
**Exposure**	30 ± 11 nM (steady-state *C*_min_)	–	–	77 ± 30 nM (steady-state *C*_min_)	24 ± 13 nM (at *t* = 2 h)	781 ± 184 nM (*C*_max_)
**Fold *in vitro* EC_50_**	<0.1	–	–	>20	>1000	>30
**BAT activity**	–	–	–	–	–	Significant increase in BAT glucose uptake, from 1 to 130 mL × SUV_mean_ × g/mL
**Energy expenditure**	24-h EE after 8 weeks did not differ from baseline	No effect on 24 h EE	Trend for stimulatory effect on 24-h EE (2.4%)	Mean change in 24-h EE upon treatment did not differ significantly between treated and placebo	Slight increase (~50 kcal/day) in 24-h EE at the highest dose	Increased resting metabolic rate by 203 ± 40 kcal/day (+13%; *p* = 0.001)
**Fasting plasma insulin**	Trend toward decrease	–	–	No significant change	No significant change	Significant increase from 5.06 (3.56–6.19) to 7.61 (6.90–8.66) (μU/mL)
**Other observations**	No change in body weight or body composition. Increase in insulin action, decrease in 24-h RQ implying 23% increase in fat oxidation. No β_1_ or β_2_ side effects (bp/heart rate)	Acid metabolite also a β_3_ partial agonist. Increases UCP1 expression in dogs (2-week study 10 mg/kg BID) (123)	Trend for increase in spontaneous physical activity	No major lipolytic or thermogenic effect but lowered triacylglycerol concentrations. No β_1_/β_2_ side effects (heart rate or tremor)	No effect on 24-h RQ or fat oxidation	BAT metabolic activity was a significant predictor of the changes in RMR. Heart rate and systolic BP increased

*MW, molecular weight; TE, target engagement; EE, energy expenditure; RQ, respiratory quotient*.

*^a^Isoproterenol response defined as 100%*.

*^b^Primary reference refers to an, at that time, yet unpublished paper from ICI*.

*^c^Claimed to be a full agonist, but only 80% TE in in vitro assay*.

Most early human trials of β_3_-AR agonists used bodyweight reduction as a clinical endpoint, to be achieved through increased EE. Data on BAT activation and metabolic parameters are scarce, with the exception being for the CL-316,243 study (124). In this study, treatment of lean healthy men for 4 weeks did not increase EE, but resulted in a 45% increase of insulin-mediated glucose disposal and a reduced 24-h respiratory quotient (24-h RQ), indicating enhanced fat oxidation. Intriguingly, CL-316,243 significantly increased fasting FFA levels in parallel with improved insulin action. These data are in line with recently published improved glucose infusion rates in T2D patients after 10 days of mild cold exposure (43). In both cases, improved insulin action preceded any significant weight loss. This improved action of insulin may be due to partitioning of FFAs toward BAT, which in turn reduces the fatty acid burden on other metabolic tissues (see Figure 1). Indeed, improved glucose uptake was seen in the skeletal muscle of T2D patients after cold exposure (43).

Similarly to CL-316,243 treatment, chronic dosing of the β_3_-AR agonists ZD7114 and ZD2079 did not significantly increase EE, although the latter compound showed a tendency toward a non-statistically significant increase of 2% (115).

Data have been reported for both acute and chronic dosing of β_3_-AR agonist L-796568 in overweight to obese men. Acutely, van Baak et al. (125) reported a significant increase (8%) in EE after 4 h for the highest dose used. However, no average increase was observed over the period of observation, 0–4 h. Chronic dosing of L-796568 for 4 weeks did not result in any significant change in EE (121). The authors discuss possible reasons for this lack of effect, e.g., declining plasma exposure levels over time, insufficient agonist-induced BAT proliferative capability of β_3_-AR-responsive tissues in humans during chronic stimulation, potential downregulation of β_3_-AR receptors, or other functional feedback. We believe that the small effect on EE observed in the acute study is another reason.

A chronic β_3_-AR agonism study (using TAK-677) in obese humans with no observed effect on EE or diabetic parameters was reported by Redman et al. (122). Whether BAT activation truly occurred after administration of compound was not carefully assessed in this study, which makes data interpretation challenging.

In contrast to the above-mentioned β_3_-AR agonism studies, Cypess et al. recently reported a ~13% increase in resting metabolic rate (RMR) upon acute treatment with high doses of β_3_-AR agonist mirabegron (41). In this study, healthy young male subjects with detectable BAT were selected in order to provide proof of concept (PoC) for β_3_-AR agonist-mediated BAT activation. Importantly, it was found that BAT metabolic activity was a significant predictor of the changes in RMR. This study also reports a weak correlation between cold- and drug-induced detectable BAT activities. Generally, such data from the same individuals are important to help put the large quantity of cold-induced BAT activation data into a drug-discovery context. It remains to be investigated if the reported energy-expenditure effect is sustained upon chronic dosing and if the efficacy of mirabegron will persist in females and other patient subpopulations, such as those with different ages and BMIs.

The failure of β_3_-AR agonists to show clinical effects on weight loss decreased interest in the mechanism as a means of treating the metabolic syndrome. However, the recent rediscovery of BAT in adult humans as well as the demonstration of functional activation of BAT by a β_3_-AR agonist may lead to a resurgent interest in β_3_-AR agonists for the treatment of metabolic disorders. The observation that improved insulin action preceded any significant weight loss upon β_3_-AR agonist treatment is particularly encouraging.

Clearly, important questions remain unanswered with respect to the role of the β_3_-AR and the clinical profile of β_3_-AR agonists. In order to make significant progress in β_3_-AR drug discovery, the lack of translation from rodent to human (i.e., receptor expression and functional differences) and from *in vitro* to *in vivo* for the human setting needs to be resolved. The clinically assessed β_3_-AR agonists exhibit structural and physicochemical property diversity; however, clinical plasma exposures relative to EC_50_ are similar for the chronic and acute studies (Table 1), and activation of BAT would therefore be expected in the chronic studies. The lack of effect on EE observed in the chronic studies may either be due to not measuring a mechanism-relevant clinical endpoint or that the patients recruited lacked sufficient BAT to lead to an effect on overall EE. The activation of pre-existing BAT by β_3_-AR agonists may require a personalized healthcare approach, unless combined with a compound or mechanism to expand the BAT depot.

Finally, cardiovascular side effects have been associated with β_3_-AR agonist treatment, usually attributed to insufficient selectivity toward the β_1_- and β_2_-ARs. However, the *in vitro* functional activity of many of the clinical compounds assessed does not explain the increase in heart rate observed – several of the compounds are functional β_1_-AR antagonists (β_1_-AR blockers), and as such could be expected to have the opposite effect to that clinically observed. The complex nature of AR biology needs to be further investigated if this mechanism is to be reconsidered as a means of activating BAT.

## Fibroblast Growth Factor 21

Fibroblast growth factor 21 (FGF21) is a member of the endocrine FGF 19/21/23 family. FGF21 protein is expressed in liver, pancreas, and adipose tissue and is regulated by fasting, ketogenic diet, low protein diet, PPARγ, and PPARα activation as well as glucagon action (126). It acts through binding to a cell-surface receptor complex composed of conventional FGF receptors (FGFR1c/2c/3c) and the co-factor β-Klotho, leading to activation of FGF receptor substrate 2α and ERK1/2 phosphorylation. While FGF receptors are ubiquitously expressed, β-Klotho expression is restricted to a few tissues, including BAT, WAT, and liver (127, 128), which are the main sites of FGF21 action. When administered pharmacologically, FGF21 enhances EE and insulin sensitivity, reduces bodyweight, glucose, and lipids, and thus has the potential to be used for treatment of the metabolic syndrome via multiple mechanisms (129–131).

Functional enhancement of existing BAT and recruitment of beige adipocytes have been hypothesized to be the mechanism behind the EE increase, bodyweight loss, and improved glucose and lipid homeostasis induced by FGF21. FGF21 expression is increased in BAT upon cold challenge (132, 133). Adipose-derived FGF21 acts in an autocrine/paracrine manner to increase expression of UCP1 and other thermogenic genes, such as *PGC1*α, *PRDM16*, *BMP8b*, and *DIO2* in iBAT and iWAT (134, 135). In neonates, FGF21 expression in liver is increased by suckling, which occurs via activation of PPARα, leading to induced BAT thermogenesis (136).

Attempts have been made to directly assess the contribution of BAT and beige adipocytes to FGF21-mediated effects. Surgical resection of iBAT in two different mouse models showed that FGF21-mediated EE increase and bodyweight reduction were retained (137, 138). However, one of the studies showed that several beige adipocyte markers, including *PPARγ*, *PRDM16*, *PGC1α*, and *CIDEA* tended toward upregulation in subcutaneous adipose tissue, and perigonadal adipose weight also was reduced (138). The authors propose that residual BAT and beige adipocytes may have compensated for the loss of iBAT. Recently, the role of UCP1 in mediating the pharmacological effects of FGF21 was assessed by treating UCP1 knockout (UCP1 KO) mice with either recombinant FGF21 or FGF21 fused with fragment crystallizable region (Fc-FGF21) (139, 140). Surprisingly, several FGF21-mediated effects were largely retained in the absence of UCP1. Again, compensatory mechanisms seem to be activated in UCP1 KO mice treated with recombinant FGF21. In FGF21-treated UCP1 KO mice, reduced food intake offset the decrease in EE and resulted in a similar bodyweight reduction to that observed in FGF21-treated wild-type mice. In addition, genes regulating fatty acid metabolism were upregulated in liver and epididymal adipose tissue, suggesting that FGF21 recruits UCP1-independent pathways in these tissues to compensate for the lack of UCP1. An intriguing observation was a two- to threefold increase in FGF21 secretion in iBAT when UCP1 KO mice were challenged by cold, suggesting that FGF21 may be one of the factors that recruit alternative thermogenic mechanisms when iBAT/UCP1 fails to generate heat. It should be noted that many previous publications have showed remodeling of the WAT in UCP1 KO mice, and that alternative thermogenic mechanisms have been discussed (141, 142).

In spite of the attractive metabolic effects of FGF21, development of FGF21-based therapeutics has encountered several technical challenges, including short *in vivo* half-life and poor biophysical properties of FGF21 protein (e.g., it is prone to aggregation). To date, three different analogs of FGF21 have advanced to concept testing in human: (130, 143, 144) (i) LY2405319 (an aggregation-resistant FGF21 analog), (ii) PF-05231023 (FGF21 linked to a Fab fragment of a scaffold antibody), and (iii) ARX-618 (PEGylated FGF21). For both LY2405319 and PF-05231023, the effects on TG (close to 50% reduction in 28 days), LDL cholesterol, and HDL cholesterol were substantial and bodyweight reduction was significant. However, the failure of FGF21 analogs to achieve clinically meaningful glucose-lowering effects was unexpected, and the mechanism behind this remains to be understood. Recently, Genentech reported a bispecific monoclonal antibody agonist that binds to both FGFR1c and β-Klotho, with sustained effects on NST, EE, and bodyweight over a 35-day period after a single administration to diet-induced obese mice (145). The effects on glucose and lipids were similar to those observed after administration of recombinant FGF21, and mostly attributed to peripheral FGF21 action as CNS exposure of the antibody was reported to be minimal. Together, these data further support the importance of BAT function in FGF21 action. As for the β_3_-AR agonist story, it may be important for future clinical concept testing to assess BAT recruitment/activation in order to understand if sufficiently high FGF21 levels have been achieved in patients. In addition, it is critical to constantly monitor potential safety concerns of this treatment principle in all studies, including bone density, growth hormone resistance, and female fertility.

## Perspectives

The understanding of BAT physiology has increased rapidly in recent years. Data generated in both rodents and humans in cold/warm acclimation studies suggest that both BAT and beige adipocytes demonstrate recruitability and plasticity, which opens the door to pharmacological intervention. As obese and T2D patients are likely to have reduced BAT function, in order to realize the full potential of NST, it may be necessary to first restore the function of “whitened” brown or beige adipocytes before activation (Figure 2). In this respect, future studies using rodent models require consideration of external temperature in order to more closely mimic human physiology. Cold acclimation for a short duration in obese and diabetic humans indicates that it is possible to enhance BAT activity and achieve an improved metabolic profile before significant bodyweight reduction occurs. The improved insulin action in the absence of bodyweight change seen in the 4-week treatment of healthy individuals by CL-316,243 (124) suggests that restoration of BAT function may at least lead to improved metabolic control in patients with T2D. Acute treatment of BAT-positive human subjects with the β_3_-AR agonist mirabegron resulted in a significant increase in EE. If this effect is sustained upon chronic dosing and without eliciting cardiovascular side effects, this mechanism may finally lead to significant weight loss.

PPARγ remains an interesting target for the recruitment of BAT or beige adipocytes, although discovery of a compound that does not impair the adrenergic sensitivity or thyroid function of the adipocyte is extremely challenging. Phenotypic screening, for example a screen based on human brown adipocyte function, may however facilitate the discovery of such a compound.

Finally, if an FGF21 analog with appropriate half-life and potency could be developed, it would enable PoC testing in humans in order to understand if the potential beneficial metabolic effects of this hormone can be realized.

## Author Contributions

XP: conception, structure and content of the text, including the figures. PG: mathematical modeling of the literature data and critical examination and interpretation of pharmacokinetic and pharmacodynamic data on the β_3_-AR agonists in clinical studies. Major contribution to the Table 1. GO’M critical review and interpretation of PPARγ and β_3_-AR agonists structure, physiochemical properties and assays used for generating the data of all compounds included in Table 1. SB: critical review of bioenergetic of cell biology, general contribution of bioscience-related text, and critical reading and editing of review.

## Conflict of Interest Statement

The authors declare that the research was conducted in the absence of any commercial or financial relationships that could be construed as a potential conflict of interest.

## References

[1] IDF DIABETES ATLAS Sixth edition (2013). Available from: https://www.idf.org/sites/default/files/EN_6E_Atlas_Full_0.pdf

[2] Cypess AM Lehman S Williams G Tal I Rodman D Goldfine AB Identification and importance of brown adipose tissue in adult humans. N Engl J Med (2009) 360:1509–17. 10.1056/NEJMoa0810780 19357406 PMC2859951

[3] Nedergaard J Bengtsson T Cannon B. Unexpected evidence for active brown adipose tissue in adult humans. Am J Physiol Endocrinol Metab (2007) 293:E444–52. 10.1152/ajpendo.00691.2006 17473055

[4] van Marken Lichtenbelt WD Vanhommerig JW Smulders NM Drossaerts JM Kemerink GJ Bouvy ND Cold-activated brown adipose tissue in healthy men. N Engl J Med (2009) 360:1500–8. 10.1056/NEJMoa0808718 19357405

[5] Virtanen KA Lidell ME Orava J Heglind M Westergren R Niemi T Functional brown adipose tissue in healthy adults. N Engl J Med (2009) 360:1518–25. 10.1056/NEJMoa0808949 19357407

[6] Betz MJ Enerback S. Human brown adipose tissue: what we have learned so far. Diabetes (2015) 64:2352–60. 10.2337/db15-0146 26050667

[7] Blondin DP Labbe SM Phoenix S Guerin B Turcotte EE Richard D Contributions of white and brown adipose tissues and skeletal muscles to acute cold-induced metabolic responses in healthy men. J Physiol (2015) 593:701–14. 10.1113/jphysiol.2014.283598 25384777 PMC4324714

[8] Chechi K Carpentier AC Richard D. Understanding the brown adipocyte as a contributor to energy homeostasis. Trends Endocrinol Metab (2013) 24:408–20. 10.1016/j.tem.2013.04.002 23711353

[9] Harms M Seale P. Brown and beige fat: development, function and therapeutic potential. Nat Med (2013) 19:1252–63. 10.1038/nm.3361 24100998

[10] Nedergaard J Cannon B. The browning of white adipose tissue: some burning issues. Cell Metab (2014) 20:396–407. 10.1016/j.cmet.2014.07.005 25127354

[11] van Marken Lichtenbelt WD Schrauwen P. Implications of nonshivering thermogenesis for energy balance regulation in humans. Am J Physiol Regul Integr Comp Physiol (2011) 301:R285–96. 10.1152/ajpregu.00652.2010 21490370

[12] Vosselman MJ van Marken Lichtenbelt WD Schrauwen P. Energy dissipation in brown adipose tissue: from mice to men. Mol Cell Endocrinol (2013) 379:43–50. 10.1016/j.mce.2013.04.017 23632102

[13] Cannon B Nedergaard J. Brown adipose tissue: function and physiological significance. Physiol Rev (2004) 84:277–359. 10.1152/physrev.00015.2003 14715917

[14] Enerback S. Human brown adipose tissue. Cell Metab (2010) 11:248–52. 10.1016/j.cmet.2010.03.008 20374955

[15] Lidell ME Betz MJ Dahlqvist Leinhard O Heglind M Elander L Slawik M Evidence for two types of brown adipose tissue in humans. Nat Med (2013) 19:631–4. 10.1038/nm.3017 23603813

[16] Frontini A Vitali A Perugini J Murano I Romiti C Ricquier D White-to-brown transdifferentiation of omental adipocytes in patients affected by pheochromocytoma. Biochim Biophys Acta (2013) 1831:950–9. 10.1016/j.bbalip.2013.02.005 23454374

[17] Ricquier D Nechad M Mory G. Ultrastructural and biochemical characterization of human brown adipose tissue in pheochromocytoma. J Clin Endocrinol Metab (1982) 54:803–7. 7061689 10.1210/jcem-54-4-803

[18] Sidossis LS Porter C Saraf MK Borsheim E Radhakrishnan RS Chao T Browning of subcutaneous white adipose tissue in humans after severe adrenergic stress. Cell Metab (2015) 22:219–27. 10.1016/j.cmet.2015.06.022 26244931 PMC4541608

[19] Seale P Bjork B Yang W Kajimura S Chin S Kuang S PRDM16 controls a brown fat/skeletal muscle switch. Nature (2008) 454:961–7. 10.1038/nature07182 18719582 PMC2583329

[20] Timmons JA Wennmalm K Larsson O Walden TB Lassmann T Petrovic N Myogenic gene expression signature establishes that brown and white adipocytes originate from distinct cell lineages. Proc Natl Acad Sci U S A (2007) 104:4401–6. 10.1073/pnas.0610615104 17360536 PMC1810328

[21] Wang Q Zhang M Xu M Gu W Xi Y Qi L Brown adipose tissue activation is inversely related to central obesity and metabolic parameters in adult human. PLoS One (2015) 10:e0123795. 10.1371/journal.pone.0123795 25894250 PMC4403996

[22] Saito M Okamatsu-Ogura Y Matsushita M Watanabe K Yoneshiro T Nio-Kobayashi J High incidence of metabolically active brown adipose tissue in healthy adult humans: effects of cold exposure and adiposity. Diabetes (2009) 58:1526–31. 10.2337/db09-0530 19401428 PMC2699872

[23] Persichetti A Sciuto R Rea S Basciani S Lubrano C Mariani S Prevalence, mass, and glucose-uptake activity of (1)(8)F-FDG-detected brown adipose tissue in humans living in a temperate zone of Italy. PLoS One (2013) 8:e63391 10.1371/journal.pone.0063391 23667608 PMC3648481

[24] Lee P Greenfield JR Ho KK Fulham MJ. A critical appraisal of the prevalence and metabolic significance of brown adipose tissue in adult humans. Am J Physiol Endocrinol Metab (2010) 299:E601–6. 10.1152/ajpendo.00298.2010 20606075

[25] Lee P Swarbrick MM Zhao JT Ho KK. Inducible brown adipogenesis of supraclavicular fat in adult humans. Endocrinology (2011) 152:3597–602. 10.1210/en.2011-1349 21791556

[26] Ouellet V Labbe SM Blondin DP Phoenix S Guerin B Haman F Brown adipose tissue oxidative metabolism contributes to energy expenditure during acute cold exposure in humans. J Clin Invest (2012) 122:545–52. 10.1172/JCI60433 22269323 PMC3266793

[27] Vijgen GH Bouvy ND Teule GJ Brans B Hoeks J Schrauwen P Increase in brown adipose tissue activity after weight loss in morbidly obese subjects. J Clin Endocrinol Metab (2012) 97:E1229–33. 10.1210/jc.2012-1289 22535970

[28] Cypess AM White AP Vernochet C Schulz TJ Xue R Sass CA Anatomical localization, gene expression profiling and functional characterization of adult human neck brown fat. Nat Med (2013) 19:635–9. 10.1038/nm.3112 23603815 PMC3650129

[29] Jespersen NZ Larsen TJ Peijs L Daugaard S Homoe P Loft A A classical brown adipose tissue mRNA signature partly overlaps with brite in the supraclavicular region of adult humans. Cell Metab (2013) 17:798–805. 10.1016/j.cmet.2013.04.011 23663743

[30] Sharp LZ Shinoda K Ohno H Scheel DW Tomoda E Ruiz L Human BAT possesses molecular signatures that resemble beige/brite cells. PLoS One (2012) 7:e49452. 10.1371/journal.pone.0049452 23166672 PMC3500293

[31] Wu J Bostrom P Sparks LM Ye L Choi JH Giang AH Beige adipocytes are a distinct type of thermogenic fat cell in mouse and human. Cell (2012) 150:366–76. 10.1016/j.cell.2012.05.016 22796012 PMC3402601

[32] Bartelt A Bruns OT Reimer R Hohenberg H Ittrich H Peldschus K Brown adipose tissue activity controls triglyceride clearance. Nat Med (2011) 17:200–5. 10.1038/nm.2297 21258337

[33] Labbe SM Caron A Bakan I Laplante M Carpentier AC Lecomte R In vivo measurement of energy substrate contribution to cold-induced brown adipose tissue thermogenesis. FASEB J (2015) 29:2046–58. 10.1096/fj.14-266247 25681456

[34] Bartelt A Heeren J. Adipose tissue browning and metabolic health. Nat Rev Endocrinol (2014) 10:24–36. 10.1038/nrendo.2013.204 24146030

[35] Seale P Conroe HM Estall J Kajimura S Frontini A Ishibashi J Prdm16 determines the thermogenic program of subcutaneous white adipose tissue in mice. J Clin Invest (2011) 121:96–105. 10.1172/JCI44271 21123942 PMC3007155

[36] Abreu-Vieira G Xiao C Gavrilova O Reitman ML. Integration of body temperature into the analysis of energy expenditure in the mouse. Mol Metab (2015) 4:461–70. 10.1016/j.molmet.2015.03.001 26042200 PMC4443293

[37] Rothwell NJ Stock MJ Whither brown fat? Biosci Rep (1986) 6:3–18. 10.1007/BF01145174 3008874

[38] Hall KD Sacks G Chandramohan D Chow CC Wang YC Gortmaker SL Quantification of the effect of energy imbalance on bodyweight. Lancet (2011) 378:826–37. 10.1016/S0140-6736(11)60812-X 21872751 PMC3880593

[39] Gennemark P Hjorth S Gabrielsson J. Modeling energy intake by adding homeostatic feedback and drug intervention. J Pharmacokinet Pharmacodyn (2015) 42:79–96. 10.1007/s10928-014-9399-4 25388764

[40] Cannon B Nedergaard J. Thermogenesis challenges the adipostat hypothesis for body-weight control. Proc Nutr Soc (2009) 68:401–7. 10.1017/S0029665109990255 19775494

[41] Cypess AM Weiner LS Roberts-Toler C Franquet Elia E Kessler SH Kahn PA Activation of human brown adipose tissue by a beta3-adrenergic receptor agonist. Cell Metab (2015) 21:33–8. 10.1016/j.cmet.2014.12.009 25565203 PMC4298351

[42] McAdam-Marx C Mukherjee J Bellows BK Unni S Ye X Iloeje U Evaluation of the relationship between weight change and glycemic control after initiation of antidiabetic therapy in patients with type 2 diabetes using electronic medical record data. Diabetes Res Clin Pract (2014) 103:402–11. 10.1016/j.diabres.2013.12.038 24503045

[43] Hanssen MJ Hoeks J Brans B van der Lans AA Schaart G van den Driessche JJ Short-term cold acclimation improves insulin sensitivity in patients with type 2 diabetes mellitus. Nat Med (2015) 21:863–5. 10.1038/nm.3891 26147760

[44] Merovci A Solis-Herrera C Daniele G Eldor R Fiorentino TV Tripathy D Dapagliflozin improves muscle insulin sensitivity but enhances endogenous glucose production. J Clin Invest (2014) 124:509–14. 10.1172/JCI70704 24463448 PMC3904617

[45] Bartesaghi S Hallen S Huang L Svensson PA Momo RA Wallin S Thermogenic activity of UCP1 in human white fat-derived beige adipocytes. Mol Endocrinol (2015) 29:130–9. 10.1210/me.2014-1295 25389910 PMC5414770

[46] Shabalina IG Petrovic N de Jong JM Kalinovich AV Cannon B Nedergaard J. UCP1 in brite/beige adipose tissue mitochondria is functionally thermogenic. Cell Rep (2013) 5:1196–203. 10.1016/j.celrep.2013.10.044 24290753

[47] Seale P. Transcriptional regulatory circuits controlling brown fat development and activation. Diabetes (2015) 64:2369–75. 10.2337/db15-0203 26050669 PMC4477361

[48] Wu J Jun H McDermott JR. Formation and activation of thermogenic fat. Trends Genet (2015) 31:232–8. 10.1016/j.tig.2015.03.003 25851693 PMC4416987

[49] Festuccia WT Blanchard PG Deshaies Y Control of brown adipose tissue glucose and lipid metabolism by PPARgamma. Front Endocrinol (2011) 2:84 10.3389/fendo.2011.00084 PMC3356105 22654830

[50] Arch JR. The discovery of drugs for obesity, the metabolic effects of leptin and variable receptor pharmacology: perspectives from beta3-adrenoceptor agonists. Naunyn Schmiedebergs Arch Pharmacol (2008) 378:225–40. 10.1007/s00210-008-0271-1 18612674

[51] Atgie C D’Allaire F Bukowiecki LJ. Role of beta1- and beta3-adrenoceptors in the regulation of lipolysis and thermogenesis in rat brown adipocytes. Am J Physiol (1997) 273:C1136–42. 9357756 10.1152/ajpcell.1997.273.4.C1136

[52] Granneman JG. Why do adipocytes make the beta 3 adrenergic receptor? Cell Signal (1995) 7:9–15. 10.1016/0898-6568(94)00066-K 7756115

[53] Lowell BB Flier JS. Brown adipose tissue, beta 3-adrenergic receptors, and obesity. Annu Rev Med (1997) 48:307–16. 10.1146/annurev.med.48.1.307 9046964

[54] Muzzin P Revelli JP Kuhne F Gocayne JD McCombie WR Venter JC An adipose tissue-specific beta-adrenergic receptor. Molecular cloning and down-regulation in obesity. J Biol Chem (1991) 266:24053–8. 1721063

[55] Carey AL Formosa MF Van Every B Bertovic D Eikelis N Lambert GW Ephedrine activates brown adipose tissue in lean but not obese humans. Diabetologia (2013) 56:147–55. 10.1007/s00125-012-2748-1 23064293

[56] Cypess AM Chen YC Sze C Wang K English J Chan O Cold but not sympathomimetics activates human brown adipose tissue in vivo. Proc Natl Acad Sci U S A (2012) 109:10001–5. 10.1073/pnas.1207911109 22665804 PMC3382513

[57] Vosselman MJ van der Lans AA Brans B Wierts R van Baak MA Schrauwen P Systemic beta-adrenergic stimulation of thermogenesis is not accompanied by brown adipose tissue activity in humans. Diabetes (2012) 61:3106–13. 10.2337/db12-0288 22872233 PMC3501890

[58] Nicholls DG. A history of UCP1. Biochem Soc Trans (2001) 29:751–5. 10.1042/bst0290751 11709069

[59] Ahmadian M Abbott MJ Tang T Hudak CS Kim Y Bruss M Desnutrin/ATGL is regulated by AMPK and is required for a brown adipose phenotype. Cell Metab (2011) 13:739–48. 10.1016/j.cmet.2011.05.002 21641555 PMC3148136

[60] Li Y Fromme T Schweizer S Schottl T Klingenspor M. Taking control over intracellular fatty acid levels is essential for the analysis of thermogenic function in cultured primary brown and brite/beige adipocytes. EMBO Rep (2014) 15:1069–76. 10.15252/embr.201438775 25135951 PMC4253847

[61] Mottillo EP Bloch AE Leff T Granneman JG Lipolytic products activate peroxisome proliferator-activated receptor (PPAR) alpha and delta in brown adipocytes to match fatty acid oxidation with supply. J Biol Chem (2012) 287:25038–48. 10.1074/jbc.M112.374041 22685301 PMC3408177

[62] Zechner R FLUX FAT: enzymes, regulators, and pathophysiology of intracellular lipolysis. EMBO Mol Med (2015) 7:359–62. 10.15252/emmm.201404846 25604059 PMC4403037

[63] Kajimura S Saito M. A new era in brown adipose tissue biology: molecular control of brown fat development and energy homeostasis. Annu Rev Physiol (2014) 76:225–49. 10.1146/annurev-physiol-021113-170252 24188710 PMC4090362

[64] Collins S Daniel KW Rohlfs EM Ramkumar V Taylor IL Gettys TW. Impaired expression and functional activity of the beta 3- and beta 1-adrenergic receptors in adipose tissue of congenitally obese (C57BL/6J ob/ob) mice. Mol Endocrinol (1994) 8:518–27. 10.1210/me.8.4.518 7914350

[65] Triandafillou J Himms-Hagen J. Brown adipose tissue in genetically obese (fa/fa) rats: response to cold and diet. Am J Physiol (1983) 244:E145–50. 6297307 10.1152/ajpendo.1983.244.2.E145

[66] Rogers NH Landa A Park S Smith RG. Aging leads to a programmed loss of brown adipocytes in murine subcutaneous white adipose tissue. Aging Cell (2012) 11:1074–83. 10.1111/acel.12010 23020201 PMC3839316

[67] Rafael J Fesser W Nicholls DG. Cold adaptation in guinea pig at level of isolated brown adipocyte. Am J Physiol (1986) 250:C228–35. 3953778 10.1152/ajpcell.1986.250.2.C228

[68] Blondin DP Labbe SM Noll C Kunach M Phoenix S Guerin B Selective impairment of glucose but not fatty acid or oxidative metabolism in brown adipose tissue of subjects with type 2 diabetes. Diabetes (2015) 64:2388–97. 10.2337/db14-1651 25677914

[69] Blondin DP Labbe SM Tingelstad HC Noll C Kunach M Phoenix S Increased brown adipose tissue oxidative capacity in cold-acclimated humans. J Clin Endocrinol Metab (2014) 99:E438–46. 10.1210/jc.2013-3901 24423363 PMC4213359

[70] Lee P Smith S Linderman J Courville AB Brychta RJ Dieckmann W Temperature-acclimated brown adipose tissue modulates insulin sensitivity in humans. Diabetes (2014) 63:3686–98. 10.2337/db14-0513 24954193 PMC4207391

[71] Rosenwald M Perdikari A Rulicke T Wolfrum C. Bi-directional interconversion of brite and white adipocytes. Nat Cell Biol (2013) 15:659–67. 10.1038/ncb2740 23624403

[72] Soccio RE Chen ER Lazar MA. Thiazolidinediones and the promise of insulin sensitization in type 2 diabetes. Cell Metab (2014) 20:573–91. 10.1016/j.cmet.2014.08.005 25242225 PMC4192012

[73] Oakes ND Thalen PG Jacinto SM Ljung B. Thiazolidinediones increase plasma-adipose tissue FFA exchange capacity and enhance insulin-mediated control of systemic FFA availability. Diabetes (2001) 50:1158–65. 10.2337/diabetes.50.5.1158 11334421

[74] Digby JE Montague CT Sewter CP Sanders L Wilkison WO O’Rahilly S Thiazolidinedione exposure increases the expression of uncoupling protein 1 in cultured human preadipocytes. Diabetes (1998) 47:138–41. 10.2337/diab.47.1.138 9421389

[75] Elabd C Chiellini C Carmona M Galitzky J Cochet O Petersen R Human multipotent adipose-derived stem cells differentiate into functional brown adipocytes. Stem Cells (2009) 27:2753–60. 10.1002/stem.200 19697348

[76] Ohno H Shinoda K Spiegelman BM Kajimura S PPARgamma agonists induce a white-to-brown fat conversion through stabilization of PRDM16 protein. Cell Metab (2012) 15:395–404. 10.1016/j.cmet.2012.01.019 22405074 PMC3410936

[77] Petrovic N Shabalina IG Timmons JA Cannon B Nedergaard J. Thermogenically competent nonadrenergic recruitment in brown preadipocytes by a PPARgamma agonist. Am J Physiol Endocrinol Metab (2008) 295:E287–96. 10.1152/ajpendo.00035.2008 18492776

[78] Sears DD Hsiao G Hsiao A Yu JG Courtney CH Ofrecio JM Mechanisms of human insulin resistance and thiazolidinedione-mediated insulin sensitization. Proc Natl Acad Sci U S A (2009) 106:18745–50. 10.1073/pnas.0903032106 19841271 PMC2763882

[79] Burkey BF Dong M Gagen K Eckhardt M Dragonas N Chen W Effects of pioglitazone on promoting energy storage, not expenditure, in brown adipose tissue of obese fa/fa Zucker rats: comparison to CL 316,243. Metabolism (2000) 49:1301–8. 10.1053/meta.2000.9524 11079820

[80] Kelly LJ Vicario PP Thompson GM Candelore MR Doebber TW Ventre J Peroxisome proliferator-activated receptors gamma and alpha mediate in vivo regulation of uncoupling protein (UCP-1, UCP-2, UCP-3) gene expression. Endocrinology (1998) 139:4920–7. 10.1210/en.139.12.4920 9832429

[81] Smith SR De Jonge L Volaufova J Li Y Xie H Bray GA. Effect of pioglitazone on body composition and energy expenditure: a randomized controlled trial. Metabolism (2005) 54:24–32. 10.1016/j.metabol.2004.07.008 15562376

[82] Sell H Berger JP Samson P Castriota G Lalonde J Deshaies Y Peroxisome proliferator-activated receptor gamma agonism increases the capacity for sympathetically mediated thermogenesis in lean and ob/ob mice. Endocrinology (2004) 145:3925–34. 10.1210/en.2004-0321 15131020

[83] Festuccia WT Blanchard PG Oliveira TB Magdalon J Paschoal VA Richard D PPARgamma activation attenuates cold-induced upregulation of thyroid status and brown adipose tissue PGC-1alpha and D2. Am J Physiol Regul Integr Comp Physiol (2012) 303:R1277–85. 10.1152/ajpregu.00299.2012 23100029 PMC3532587

[84] Festuccia WT Blanchard PG Turcotte V Laplante M Sariahmetoglu M Brindley DN The PPARgamma agonist rosiglitazone enhances rat brown adipose tissue lipogenesis from glucose without altering glucose uptake. Am J Physiol Regul Integr Comp Physiol (2009) 296:R1327–35. 10.1152/ajpregu.91012.2008 19211718

[85] Bakopanos E Silva JE. Thiazolidinediones inhibit the expression of beta3-adrenergic receptors at a transcriptional level. Diabetes (2000) 49:2108–15. 10.2337/diabetes.49.12.2108 11118014

[86] Festuccia WT Oztezcan S Laplante M Berthiaume M Michel C Dohgu S Peroxisome proliferator-activated receptor-gamma-mediated positive energy balance in the rat is associated with reduced sympathetic drive to adipose tissues and thyroid status. Endocrinology (2008) 149:2121–30. 10.1210/en.2007-1553 18218698

[87] Bogacka I Ukropcova B McNeil M Gimble JM Smith SR. Structural and functional consequences of mitochondrial biogenesis in human adipocytes in vitro. J Clin Endocrinol Metab (2005) 90:6650–6. 10.1210/jc.2005-1024 16204368

[88] Bogacka I Gettys TW de Jonge L Nguyen T Smith JM Xie H The effect of beta-adrenergic and peroxisome proliferator-activated receptor-gamma stimulation on target genes related to lipid metabolism in human subcutaneous adipose tissue. Diabetes Care (2007) 30:1179–86. 10.2337/dc06-1962 17351280

[89] Nettles KW Greene GL. Ligand control of coregulator recruitment to nuclear receptors. Annu Rev Physiol (2005) 67:309–33. 10.1146/annurev.physiol.66.032802.154710 15709961

[90] Lehmann JM Moore LB Smith-Oliver TA Wilkison WO Willson TM Kliewer SA An antidiabetic thiazolidinedione is a high affinity ligand for peroxisome proliferator-activated receptor gamma (PPAR gamma). J Biol Chem (1995) 270:12953–6. 10.1074/jbc.270.22.12953 7768881

[91] Cho N Momose Y. Peroxisome proliferator-activated receptor gamma agonists as insulin sensitizers: from the discovery to recent progress. Curr Top Med Chem (2008) 8:1483–507. 10.2174/156802608786413474 19075761

[92] Henke BR 1. Peroxisome proliferator-activated receptor gamma (PPARgamma) ligands and their therapeutic utility. Prog Med Chem (2004) 42:1–53. 10.1016/S0079-6468(04)42001-3 15003718

[93] Nevin DK Lloyd DG Fayne D. Rational targeting of peroxisome proliferating activated receptor subtypes. Curr Med Chem (2011) 18:5598–623. 10.2174/092986711798347243 22172067

[94] Savkur RS Miller AR. Investigational PPAR-gamma agonists for the treatment of Type 2 diabetes. Expert Opin Investig Drugs (2006) 15:763–78. 10.1517/13543784.15.7.763 16787140

[95] Takada I Makishima M PPARgamma ligands and their therapeutic applications: a patent review (2008-2014). Expert Opin Ther Pat (2015) 25:175–91. 10.1517/13543776.2014.985206 25416646

[96] Willson TM Brown PJ Sternbach DD Henke BR The PPARs: from orphan receptors to drug discovery. J Med Chem (2000) 43:527–50. 10.1021/jm990554g 10691680

[97] Garcia-Vallve S Guasch L Tomas-Hernandez S Del Bas JM Ollendorff V Arola L Peroxisome proliferator-activated receptor gamma (PPARgamma) and ligand choreography: newcomers take the stage. J Med Chem (2015) 58:5381–94. 10.1021/jm501155f 25734377

[98] Abbas A Blandon J Rude J Elfar A Mukherjee D PPAR-gamma agonist in treatment of diabetes: cardiovascular safety considerations. Cardiovasc Hematol Agents Med Chem (2012) 10:124–34. 10.2174/187152512800388948 22471957

[99] Bortolini M Wright MB Bopst M Balas B. Examining the safety of PPAR agonists – current trends and future prospects. Expert Opin Drug Saf (2013) 12:65–79. 10.1517/14740338.2013.741585 23134541

[100] Rubenstrunk A Hanf R Hum DW Fruchart JC Staels B. Safety issues and prospects for future generations of PPAR modulators. Biochim Biophys Acta (2007) 1771:1065–81. 10.1016/j.bbalip.2007.02.003 17428730

[101] Tang WH Maroo A. PPARgamma agonists: safety issues in heart failure. Diabetes Obes Metab (2007) 9:447–54. 10.1111/j.1463-1326.2006.00616.x 17587386

[102] Germain P Iyer J Zechel C Gronemeyer H. Co-regulator recruitment and the mechanism of retinoic acid receptor synergy. Nature (2002) 415:187–92. 10.1038/415187a 11805839

[103] Hamza MS Pott S Vega VB Thomsen JS Kandhadayar GS Ng PW De-novo identification of PPARgamma/RXR binding sites and direct targets during adipogenesis. PLoS One (2009) 4:e4907. 10.1371/journal.pone.0004907 19300518 PMC2654672

[104] Bulynko YA O’Malley BW. Nuclear receptor coactivators: structural and functional biochemistry. Biochemistry (2011) 50:313–28. 10.1021/bi101762x 21141906 PMC3647688

[105] Lonard DM O’Malley BW. Nuclear receptor coregulators: judges, juries, and executioners of cellular regulation. Mol Cell (2007) 27:691–700. 10.1016/j.molcel.2007.08.012 17803935

[106] Millard CJ Watson PJ Fairall L Schwabe JW. An evolving understanding of nuclear receptor coregulator proteins. J Mol Endocrinol (2013) 51:T23–36. 10.1530/JME-13-0227 24203923 PMC3963257

[107] Nolte RT Wisely GB Westin S Cobb JE Lambert MH Kurokawa R Ligand binding and co-activator assembly of the peroxisome proliferator-activated receptor-gamma. Nature (1998) 395:137–43. 10.1038/25931 9744270

[108] Ahmadian M Suh JM Hah N Liddle C Atkins AR Downes M PPARgamma signaling and metabolism: the good, the bad and the future. Nat Med (2013) 19:557–66. 10.1038/nm.3159 23652116 PMC3870016

[109] Banks AS McAllister FE Camporez JP Zushin PJ Jurczak MJ Laznik-Bogoslavski D An ERK/Cdk5 axis controls the diabetogenic actions of PPARgamma. Nature (2015) 517:391–5. 10.1038/nature13887 25409143 PMC4297557

[110] Moisan A Lee YK Zhang JD Hudak CS Meyer CA Prummer M White-to-brown metabolic conversion of human adipocytes by JAK inhibition. Nat Cell Biol (2015) 17:57–67. 10.1038/ncb3075 25487280 PMC4276482

[111] Dolan JA Muenkel HA Burns MG Pellegrino SM Fraser CM Pietri F Beta-3 adrenoceptor selectivity of the dioxolane dicarboxylate phenethanolamines. J Pharmacol Exp Ther (1994) 269:1000–6. 7912272

[112] Emorine LJ Marullo S Briend-Sutren MM Patey G Tate K Delavier-Klutchko C Molecular characterization of the human beta 3-adrenergic receptor. Science (1989) 245:1118–21. 10.1126/science.2570461 2570461

[113] Lelias JM Kaghad M Rodriguez M Chalon P Bonnin J Dupre I Molecular cloning of a human beta 3-adrenergic receptor cDNA. FEBS Lett (1993) 324:127–30. 10.1016/0014-5793(93)81377-C 8389717

[114] Hu B Ellingboe J Han S Largis E Lim K Malamas M Novel (4-piperidin-1-yl)-phenyl sulfonamides as potent and selective human beta(3) agonists. Bioorg Med Chem (2001) 9:2045–59. 10.1016/S0968-0896(01)00114-6 11504641

[115] Buemann B Toubro S Astrup A Effects of the two beta3-agonists, ZD7114 and ZD2079 on 24 hour energy expenditure and respiratory quotient in obese subjects. Int J Obes Relat Metab Disord (2000) 24:1553–60. 10.1038/sj.ijo.0801452 11126205

[116] Pietri-Rouxel F Strosberg AD. Pharmacological characteristics and species-related variations of beta 3-adrenergic receptors. Fundam Clin Pharmacol (1995) 9:211–8. 10.1111/j.1472-8206.1995.tb00288.x 7557816

[117] Mathvink RJ Tolman JS Chitty D Candelore MR Cascieri MA Colwell LF Jr Discovery of a potent, orally bioavailable beta(3) adrenergic receptor agonist, (R)-N-[4-[2-[[2-hydroxy-2-(3-pyridinyl)ethyl]amino]ethyl]phenyl]-4-[4 -[4-(trifluoromethyl)phenyl]thiazol-2-yl]benzenesulfonamide. J Med Chem (2000) 43:3832–6. 10.1021/jm000286i 11052788

[118] Harada H Hirokawa Y Suzuki K Hiyama Y Oue M Kawashima H Discovery of a novel and potent human and rat beta3-adrenergic receptor agonist, [3-[(2R)-[[(2R)-(3-chlorophenyl)-2-hydroxyethyl]amino]propyl]-1H-indol-7-yloxy]acetic acid. Chem Pharm Bull (2005) 53:184–98. 10.1248/cpb.53.184 15684518

[119] Takasu T Ukai M Sato S Matsui T Nagase I Maruyama T Effect of (R)-2-(2-aminothiazol-4-yl)-4’-{2-[(2-hydroxy-2-phenylethyl)amino]ethyl} acetanilide (YM178), a novel selective beta3-adrenoceptor agonist, on bladder function. J Pharmacol Exp Ther (2007) 321:642–7. 10.1124/jpet.106.115840 17293563

[120] Blin N Nahmias C Drumare MF Strosberg AD. Mediation of most atypical effects by species homologues of the beta 3-adrenoceptor. Br J Pharmacol (1994) 112:911–9. 10.1111/j.1476-5381.1994.tb13167.x 7921620 PMC1910199

[121] Larsen TM Toubro S van Baak MA Gottesdiener KM Larson P Saris WH Effect of a 28-d treatment with L-796568, a novel beta(3)-adrenergic receptor agonist, on energy expenditure and body composition in obese men. Am J Clin Nutr (2002) 76:780–8. 12324291 10.1093/ajcn/76.4.780

[122] Redman LM de Jonge L Fang X Gamlin B Recker D Greenway FL Lack of an effect of a novel beta3-adrenoceptor agonist, TAK-677, on energy metabolism in obese individuals: a double-blind, placebo-controlled randomized study. J Clin Endocrinol Metab (2007) 92:527–31. 10.1210/jc.2006-1740 17118998

[123] Champigny O Ricquier D Blondel O Mayers RM Briscoe MG Holloway BR. Beta 3-adrenergic receptor stimulation restores message and expression of brown-fat mitochondrial uncoupling protein in adult dogs. Proc Natl Acad Sci U S A (1991) 88:10774–7. 10.1073/pnas.88.23.10774 1720550 PMC53013

[124] Weyer C Tataranni PA Snitker S Danforth E Jr Ravussin E. Increase in insulin action and fat oxidation after treatment with CL 316,243, a highly selective beta3-adrenoceptor agonist in humans. Diabetes (1998) 47:1555–61. 10.2337/diabetes.47.10.1555 9753292

[125] van Baak MA Hul GB Toubro S Astrup A Gottesdiener KM DeSmet M Acute effect of L-796568, a novel beta 3-adrenergic receptor agonist, on energy expenditure in obese men. Clin Pharmacol Ther (2002) 71:272–9. 10.1067/mcp.2002.122527 11956510

[126] Laeger T Henagan TM Albarado DC Redman LM Bray GA Noland RC FGF21 is an endocrine signal of protein restriction. J Clin Invest (2014) 124:3913–22. 10.1172/JCI74915 25133427 PMC4153701

[127] Adams AC Cheng CC Coskun T Kharitonenkov A FGF21 requires betaklotho to act in vivo. PLoS One (2012) 7:e49977 10.1371/journal.pone.0049977 23209629 PMC3507945

[128] Ding X Boney-Montoya J Owen BM Bookout AL Coate KC Mangelsdorf DJ betaKlotho is required for fibroblast growth factor 21 effects on growth and metabolism. Cell Metab (2012) 16:387–93. 10.1016/j.cmet.2012.08.002 22958921 PMC3447537

[129] Angelin B Larsson TE Rudling M Circulating fibroblast growth factors as metabolic regulators – a critical appraisal. Cell Metab (2012) 16:693–705. 10.1016/j.cmet.2012.11.001 23217254

[130] Gimeno RE Moller DE FGF21-based pharmacotherapy – potential utility for metabolic disorders. Trends Endocrinol Metab (2014) 25:303–11. 10.1016/j.tem.2014.03.001 24709036

[131] Owen BM Ding X Morgan DA Coate KC Bookout AL Rahmouni K FGF21 acts centrally to induce sympathetic nerve activity, energy expenditure, and weight loss. Cell Metab (2014) 20:670–7. 10.1016/j.cmet.2014.07.012 25130400 PMC4192037

[132] Chartoumpekis DV Habeos IG Ziros PG Psyrogiannis AI Kyriazopoulou VE Papavassiliou AG. Brown adipose tissue responds to cold and adrenergic stimulation by induction of FGF21. Mol Med (2011) 17:736–40. 10.2119/molmed.2011.00075 21373720 PMC3146611

[133] Hanssen MJ Broeders E Samms RJ Vosselman MJ van der Lans AA Cheng CC Serum FGF21 levels are associated with brown adipose tissue activity in humans. Sci Rep (2015) 5:10275. 10.1038/srep10275 25985218 PMC4434994

[134] Fisher FM Kleiner S Douris N Fox EC Mepani RJ Verdeguer F FGF21 regulates PGC-1alpha and browning of white adipose tissues in adaptive thermogenesis. Genes Dev (2012) 26:271–81. 10.1101/gad.177857.111 22302939 PMC3278894

[135] Lee P Linderman JD Smith S Brychta RJ Wang J Idelson C Irisin and FGF21 are cold-induced endocrine activators of brown fat function in humans. Cell Metab (2014) 19:302–9. 10.1016/j.cmet.2013.12.017 24506871 PMC7647184

[136] Hondares E Gallego-Escuredo JM Flachs P Frontini A Cereijo R Goday A Fibroblast growth factor-21 is expressed in neonatal and pheochromocytoma-induced adult human brown adipose tissue. Metabolism (2014) 63:312–7. 10.1016/j.metabol.2013.11.014 24369918

[137] Camporez JP Jornayvaz FR Petersen MC Pesta D Guigni BA Serr J Cellular mechanisms by which FGF21 improves insulin sensitivity in male mice. Endocrinology (2013) 154:3099–109. 10.1210/en.2013-1191 23766126 PMC3749479

[138] Emanuelli B Vienberg SG Smyth G Cheng C Stanford KI Arumugam M Interplay between FGF21 and insulin action in the liver regulates metabolism. J Clin Invest (2015) 125:458 10.1172/JCI80223 25654556 PMC4382261

[139] Samms RJ Smith DP Cheng CC Antonellis PP Perfield JW II Kharitonenkov A Discrete aspects of FGF21 in vivo pharmacology do not require UCP1. Cell Rep (2015) 11:991–9. 10.1016/j.celrep.2015.04.046 25956583

[140] Veniant MM Sivits G Helmering J Komorowski R Lee J Fan W Pharmacologic effects of FGF21 are independent of the “browning” of white adipose tissue. Cell Metab (2015) 21:731–8. 10.1016/j.cmet.2015.04.019 25955208

[141] Keipert S Kutschke M Lamp D Brachthauser L Neff F Meyer CW Genetic disruption of uncoupling protein 1 in mice renders brown adipose tissue a significant source of FGF21 secretion. Mol Metab (2015) 4:537–42. 10.1016/j.molmet.2015.04.006 26137441 PMC4481421

[142] Ukropec J Anunciado RP Ravussin Y Hulver MW Kozak LP. UCP1-independent thermogenesis in white adipose tissue of cold-acclimated Ucp1-/- mice. J Biol Chem (2006) 281:31894–908. 10.1074/jbc.M606114200 16914547

[143] Dong JQ Rossulek M Somayaji VR Baltrukonis D Liang Y Hudson K Pharmacokinetics and pharmacodynamics of PF-05231023, a novel long-acting FGF21 mimetic, in a first-in-human study. Br J Clin Pharmacol (2015) 80(5):1051–63. 10.1111/bcp.12676 25940675 PMC4631178

[144] Gaich G Chien JY Fu H Glass LC Deeg MA Holland WL The effects of LY2405319, an FGF21 analog, in obese human subjects with type 2 diabetes. Cell Metab (2013) 18:333–40. 10.1016/j.cmet.2013.08.005 24011069

[145] Kolumam G Chen MZ Tong R Zavala-Solorio J Kates L van Bruggen N Sustained brown fat stimulation and insulin sensitization by a humanized bispecific antibody agonist for fibroblast growth factor receptor 1/betaKlotho complex. EBioMedicine (2015) 2:730–43. 10.1016/j.ebiom.2015.05.028 26288846 PMC4534681

